# Monoubiquitination and Activity of the Paracaspase MALT1 Requires Glutamate 549 in the Dimerization Interface

**DOI:** 10.1371/journal.pone.0072051

**Published:** 2013-08-19

**Authors:** Katrin Cabalzar, Christiane Pelzer, Annette Wolf, Georg Lenz, Justyna Iwaszkiewicz, Vincent Zoete, Stephan Hailfinger, Margot Thome

**Affiliations:** 1 Department of Biochemistry, University of Lausanne, Epalinges, Switzerland; 2 Department of Hematology, Oncology and Tumor Immunology, Molecular Cancer Research Center, Charité - Universitätsmedizin Berlin, Germany; 3 Swiss Institute of Bioinformatics, Lausanne, Switzerland; J. Heyrovsky Institute of Physical Chemistry, Czech Republic

## Abstract

The mucosa-associated lymphoid tissue protein-1 (MALT1, also known as paracaspase) is a protease whose activity is essential for the activation of lymphocytes and the growth of cells derived from human diffuse large B-cell lymphomas of the activated B-cell subtype (ABC DLBCL). Crystallographic approaches have shown that MALT1 can form dimers via its protease domain, but why dimerization is relevant for the biological activity of MALT1 remains largely unknown. Using a molecular modeling approach, we predicted Glu 549 (E549) to be localized within the MALT1 dimer interface and thus potentially relevant. Experimental mutation of this residue into alanine (E549A) led to a complete impairment of MALT1 proteolytic activity. This correlated with an impaired capacity of the mutant to form dimers of the protease domain *in vitro*, and a reduced capacity to promote NF-κB activation and transcription of the growth-promoting cytokine interleukin-2 in antigen receptor-stimulated lymphocytes. Moreover, this mutant could not rescue the growth of ABC DLBCL cell lines upon MALT1 silencing. Interestingly, the MALT1 mutant E549A was unable to undergo monoubiquitination, which we identified previously as a critical step in MALT1 activation. Collectively, these findings suggest a model in which E549 at the dimerization interface is required for the formation of the enzymatically active, monoubiquitinated form of MALT1.

## Introduction

The protease MALT1 (also known as paracaspase) plays a central role in the antigen receptor-mediated activation of lymphocytes and the pathogenesis of human diffuse large B-cell lymphoma (DLBCL) of the activated B-cell (ABC) subtype [1], [2]. In resting naïve lymphocytes, MALT1 is present in its catalytically inactive form, constitutively associated with the adaptor protein BCL10 [3], [4]. Upon antigen receptor triggering, MALT1 and BCL10 form a complex with the scaffold protein CARMA1 (also known as CARD11) [5], [6] that promotes the activation of the transcription factor nuclear factor kappa B (NF-κB). NF-κB drives the expression of genes that promote the proliferation and survival of the activated lymphocytes [7]. In resting lymphocytes, NF-κB complexes are present mainly as p50-RelA and p50-cRel heterodimers [8]. These are kept inactive by inhibitor of kappa B (IκB) proteins, which retain NF-κB heterodimers in the cytoplasm [9], but also by the NF-κB family member RelB, which acts as an NF-κB inhibitor in lymphocytes by binding RelA and c-Rel and preventing their DNA binding [10]–[13]. MALT1 promotes NF-κB activation by both its scaffold and its enzymatic function [1], [2]. As a scaffold, MALT1 promotes the recruitment of the ubiquitin ligase TRAF6 and the subsequent ubiquitination-dependent recruitment and activation of the IκB kinase (IKK) complex, which phosphorylates and thereby initiates the degradation of the NF-κB inhibitor IκBα [14]–[18]. As an enzyme with protease activity, MALT1 also promotes NF-κB activation by cleaving the inhibitor RelB, which is subsequently degraded by the proteasome [10]. In addition, MALT1 promotes lymphocyte activation by cleaving A20 and CYLD, deubiquitinating enzymes that have inhibitory roles in the NF-κB and JNK pathway, respectively [19], [20], and by cleavage of BCL10, which promotes lymphocyte adhesion [21]. Unlike caspases, MALT1 preferentially cleaves its substrate after an arginine residue [1], [2], [22]. Consequently, inhibition of MALT1 with the arginine-based peptide inhibitor z-VRPR-fmk leads to a significant reduction in antigen-receptor mediated lymphocyte activation [21].

Recently, the protease activity of MALT1 has received particular attention as a drug target for the treatment of ABC DLBCL, a particularly aggressive form of human B-cell lymphoma that is dependent on the oncogenic activation of the CARMA1-BCL10-MALT1 pathway [23]–[25] and on the protease activity of MALT1 [26]–[29]. Indeed, inhibition of the protease activity of MALT1 with a peptide inhibitor or small molecule drugs efficiently inhibits the growth of cells derived from ABC DLBCL *in vitro* and in xenograft models [26]–[29]. Constitutive MALT1 activity may also be a driving force for the growth of B-cell lymphomas of the mucosa-associated lymphoid tissue (MALT lymphomas), which frequently have a chromosomal translocation that leads to the formation of an oncogenic fusion protein of MALT1 with the apoptosis inhibitor c-IAP2 [30]. The resulting c-IAP2-MALT1 fusion protein specifically cleaves the Ser/Thr kinase NIK and thereby promotes the oncogenic activation of the alternative NF-κB pathway [31].

Size exclusion chromatography and protein crystallography, performed in the presence or absence of the irreversible peptide inhibitor z-VRPR-fmk, show that binding to this substrate analog promotes the formation of MALT1 dimers that adopt the active conformation [32], [33]. In solution, MALT1 dimerization is favored by binding to its inhibitor [33]. When crystallized in the absence of the inhibitor, MALT1 already forms a dimer in which the active site Cys 464 (C464) adopts an inactive conformation, unable to form the catalytically active dyad with His 415 (H415) [32]. In this inactive conformation, the protease domain interacts via hydrophobic residues with the adjacent C-terminal immunoglobulin domain (Ig3). Formation of the active dimeric conformation seems to be controlled by a conformational change that alters the interaction of the protease domain with the Ig3 domain [32]. Nevertheless, the exact mechanisms by which the dimerization and activation of MALT1 are controlled *in vivo* remain poorly understood. We recently demonstrated that MALT1 is activated by monoubiquitination on a Lys residue (K644) that is situated in a structurally undefined loop within the C-terminal Ig3 domain [34]. Monoubiquitination of MALT1 is thought to favor or stabilize the active MALT1 dimer, since C-terminal fusion of a monoubiquitin moiety to MALT1 generates a constitutively active form of MALT1 that is preferentially dimeric [34]. These data, together with the crystallographic data, support the idea that MALT1 is active as a dimer, but it remains unknown how dimerization controls the catalytic and biological activity of MALT1.

Here, we show that a Glu residue (E549) localized within the dimerization interface of the MALT1 protease domain was critical for the *in vitro* dimerization of the MALT1 protease domain. Mutation of E549 into alanine (E549A) led to complete loss of the enzymatic activity of MALT1, and to a consequent loss of the growth-promoting function of MALT1 in lymphocytes and lymphoma cells. Moreover, the mutant was unable to undergo monoubiquitination, and its activity could not be restored by artificial monoubiquitination-induced dimerization. Collectively, these findings support the idea that E549 within the dimerization interface of MALT1 plays a critical role in the regulation of the enzymatic and biological activity of MALT1.

## Results

The MALT1 protease domain has sequence similarity with caspases [4] that have been shown to form catalytically active dimers [35]. To assess whether MALT1 is able to form dimers, we initially modeled the three-dimensional structure of the MALT1 protease domain based on the published structures of caspase-9, -3 and -8. The validity of the resulting model of a caspase-like domain of MALT1 was recently confirmed by published crystallographic structures of MALT1 [32], [33]. Except for the N-terminal beta strand, all secondary structural elements were predicted correctly. The Root Mean Square Deviation (RMSD) of all Cα atoms between our model and the structure of the inhibitor-bound MALT1 (3UOA.pdb, [33]) is 4.3 Å (without N-terminus and longest loops), while the RMSD of the central beta strands and the alpha helices in the dimerization interface is below 2 Å (**Fig. S1**), which confirms the general good quality of our predictions. In the model of the MALT1 dimer, we noticed that the potential dimerization interface of the MALT1 protease domain lacked the hydrophobic residues that were previously described to stabilize the caspase-8 dimer [36]. Instead, by visual inspection of the model, we noticed the presence of charged residues, Glu 549 (E549) and Arg 551 (R551), which could potentially form a salt bridge stabilizing the dimer (**Fig. S2**). These residues are invariant across species [33], and we hypothesized that mutation of these into uncharged alanine residues (E549A and R551A, respectively) might affect MALT1 dimerization and activity.

**Figure 1:**
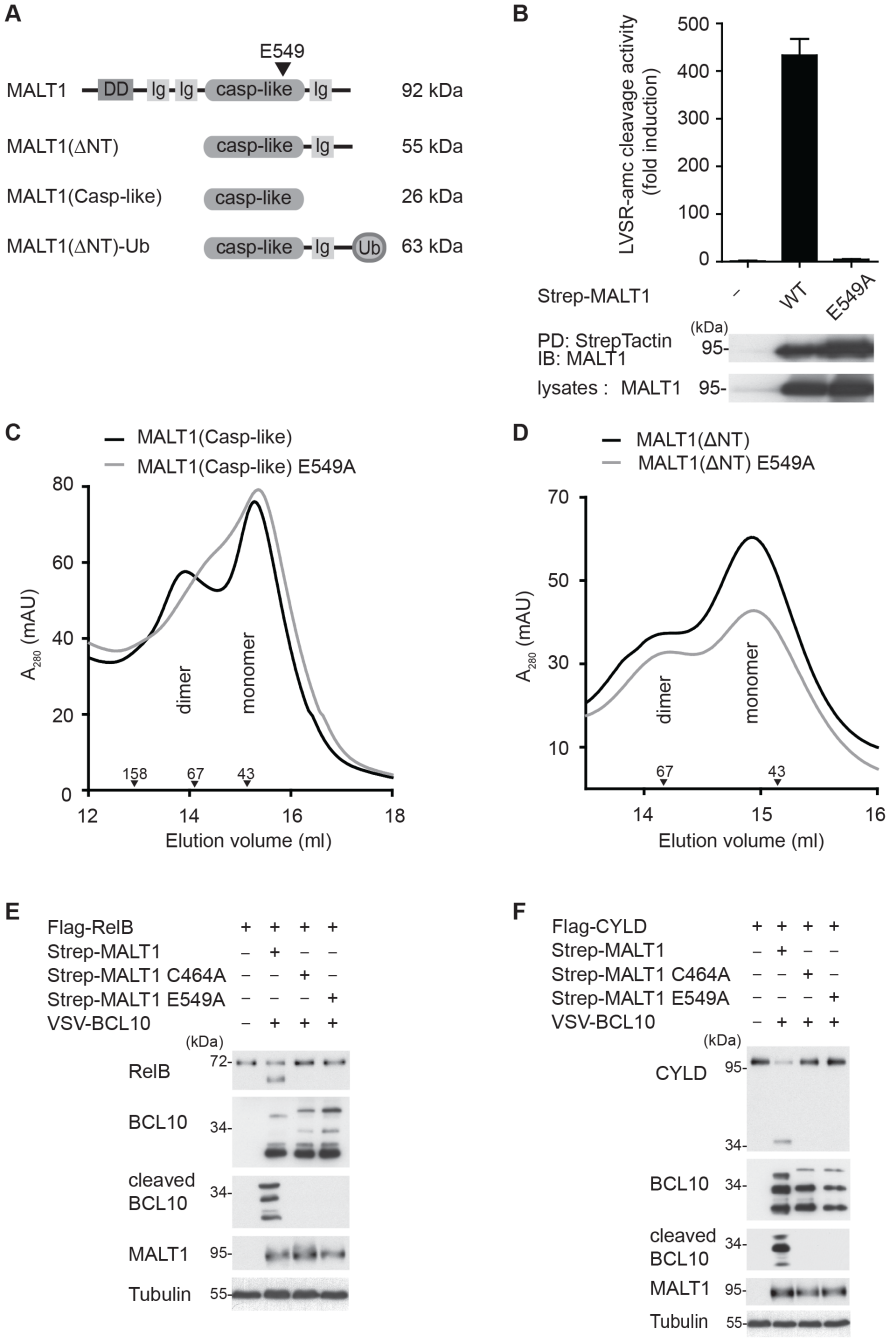
Mutation of E549 affects dimerization of the isolated protease domain and the catalytic activity of MALT1. (A) Schematic representation of the constructs used in this study. (B) The activity of the indicated Strep-tagged MALT1 wildtype (WT) and E549A mutant constructs, precipitated from transfected HEK293T cells, was determined *in vitro* in presence of 1 M ammonium citrate using the MALT1 substrate LVSR-amc. Protein levels in lysates and precipitations were controlled by immunoblot. PD: pull-down. Left margin, molecular size marker in kilodalton (kDa). (C, D) Recombinant wildtype or E549A-mutant MALT1 proteins corresponding to the isolated protease domain (aa 333–566, MALT1(Casp-like)) (C) or to the protease domain and the C-terminal extension (aa 333–824, MALT1(ΔNT)) (D) were analyzed by size exclusion chromatography in presence of PBS and 10% glycerol, presented as the absorbance at 280 nm (A_280_) in ‘milli-arbitrary units’ (mAU). Downward arrowheads indicate positions of the molecular weight standards aldolase (158 kDa), bovine serum albumin (67 kDa) and ovalbumin (43 kDa). (E, F) Strep-tagged MALT1 or its E549A mutant were co-expressed with VSV-tagged BCL10, FLAG-tagged RelB or FLAG-tagged CYLD in HEK293T cells, as indicated, and protein cleavage was assessed by immunoblot analysis of lysates. Tubulin served as a loading control throughout. Data are representative of two (B, D, E and F) or three independent (C) experiments.

To test this hypothesis, we expressed different MALT1 constructs (**Fig. 1A**) in HEK293T cells and assessed the precipitated proteins for their catalytic activity *in vitro* in the presence of the kosmotropic salt ammonium citrate, which is known to activate initiator caspases by favoring their dimerization [37], [38]. As previously reported, wildtype MALT1 was highly active in ammonium citrate buffer [19], [22], [34], but this activity was completely lost in the E549A mutant (**Fig. 1B**). The R551A mutant, on the other hand, had only a partial effect on MALT1 activity (**Fig. S3**), therefore we focused on the E549A mutant for further analysis. Using a bacterial expression system, we then generated purified recombinant constructs containing only the wildtype or E549A mutant MALT1 protease domain (Casp-like constructs), or the protease domain together with the C-terminal extension (ΔNT constructs) (**Fig. 1A**). Analysis of these constructs by size exclusion chromatography, under conditions of physiological salt concentrations, showed that wildtype MALT1 had some tendency to spontaneously form dimers in solution, consistent with previous reports [32], [39] (**Fig. 1C**). Dimerization of the constructs containing only the protease domain was affected in the E549A mutant (**Fig. 1C**), but not in the active site mutant C464A (data not shown). Impairment of dimerization was no longer apparent when a construct containing the protease domain and the C-terminal extension of MALT1 was used (**Fig. 1D**), suggesting that the C-terminal region stabilizes the dimer conformation.

Nevertheless, the E549A mutation resulted in the complete loss of MALT1 catalytic activity towards its substrates BCL10, RelB or CYLD when co-expressed in HEK293T cells (**Fig. 1** **, E and F**). However, this mutation did not affect the capacity of MALT1 to bind to BCL10 (**Fig. S4**). Thus, mutation of E549, which is part of the dimerization interface, compromises the capacity of the isolated MALT1 protease domain to form dimers and results in loss of catalytic activity of full length MALT1 *in vitro* and in living cells.

**Figure 2:**
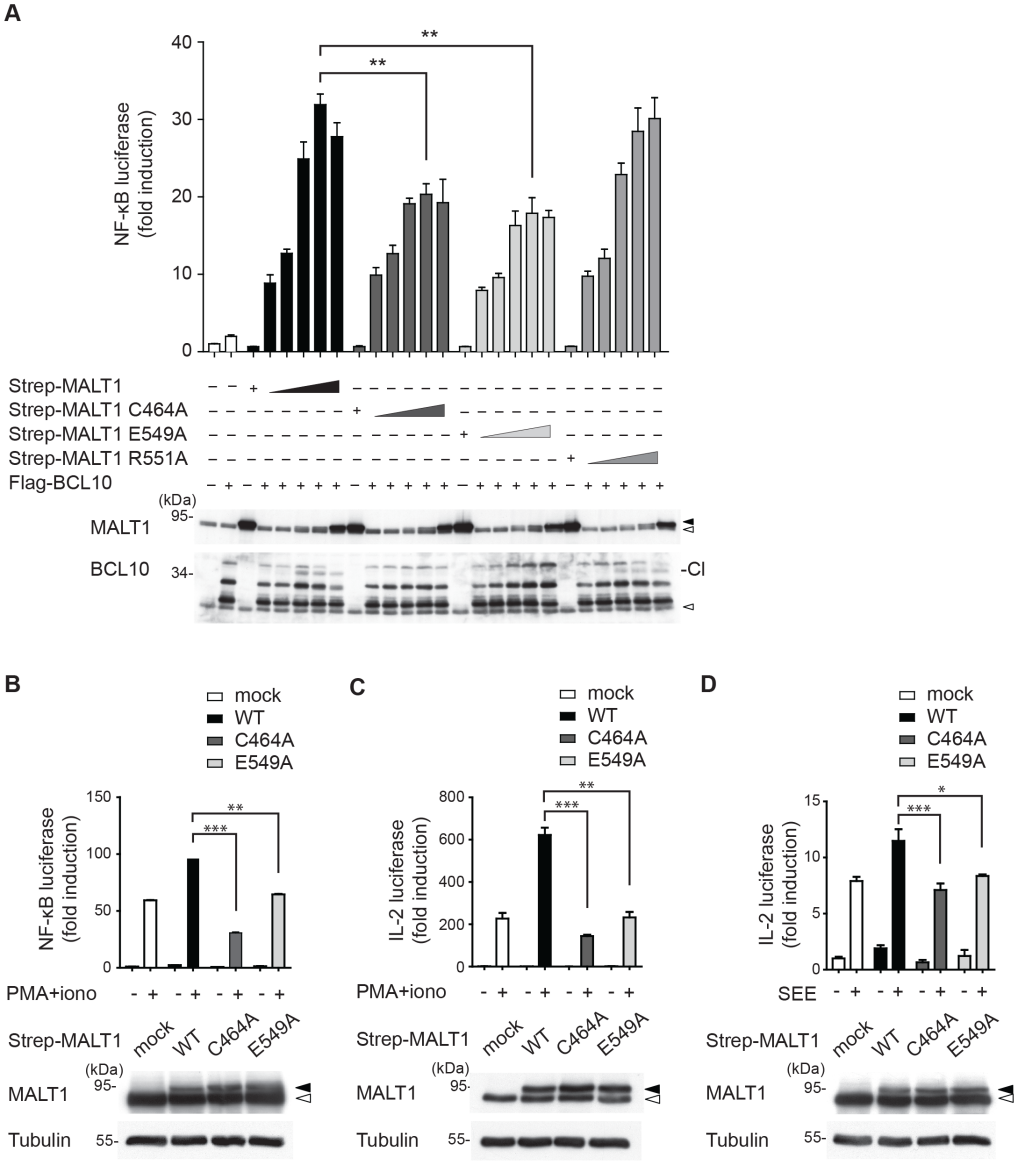
The residue E549 is required for the biological activity of MALT1 in T cells. (A) Strep-tagged MALT1 wildtype, its C464A or E549A mutant were co-expressed with FLAG-tagged BCL10 and an NF-κB reporter construct in HEK293T cells, and NF-κB induction was assessed by dual luciferase assay. The open and filled arrowheads indicate the positions of endogenous and transfected proteins, respectively. The position of the cleaved BCL10 (Cl) proteins is indicated. Statistical significance is indicated by stars. *≤0.05, **≤0.01, ***≤0.005 (two-tailed Student’s *t*-test). (B) Jurkat cells stably expressing an NF-κB luciferase reporter construct were transfected with indicated Strep-tagged MALT1 constructs, and luciferase reporter activity was measured upon T-cell stimulation by PMA and ionomycin (PMA+iono) for 16 h. (C) Jurkat cells stably expressing the SV40 large T antigen (JTag cells) were co-transfected with indicated Strep-tagged MALT1 constructs and an IL-2 reporter plasmid, and luciferase reporter activity was measured upon T-cell stimulation by PMA and ionomycin (PMA+iono) for 16 h. (D) Jurkat cells were co-transfected with indicated Strep-tagged MALT1 constructs and an IL-2 reporter plasmid, and luciferase reporter activity was measured upon T-cell stimulation with Raji B cells in the presence (+) or absence (−) of the superantigen staphylococcus enterotoxin E (SEE) for 16 h. Data are representative of three (A–C) or two (D) independent experiments.

To test the biological relevance of E549-dependent MALT1 activity, we next expressed increasing amounts of wildtype MALT1, the catalytically inactive mutant C464A or the E549A mutant together with BCL10 in HEK293T cells, and analyzed the capacity of these MALT1 constructs to promote NF-κB activation (**Fig. 2A**). In HEK293T cells, MALT1 overexpression promotes NF-κB activation mainly by its scaffold function, and only partially via its proteolytic activity [21]. Compared to wildtype MALT1, the mutant E549A had a partially impaired capacity to promote NF-κB activation in HEK293T cells (**Fig. 2A**), similar to a catalytically inactive mutant of MALT1 in which the conserved cysteine residue C464 of the catalytic site has been mutated into alanine (MALT1 C464A) [21]. No inhibitory effect on NF-κB activation, and only a minimal effect on BCL10 cleavage were observed for the R551A mutant (**Fig. 2A**). In T cells, the MALT1 protease activity controls the T-cell receptor mediated NF-κB activation by inducing the cleavage and subsequent degradation of RelB, which acts as an inhibitor of NF-κB1 in these cells [10]–[13], [40]. We analyzed the effect of the E549A mutant on antigen receptor-induced activation of the NF-κB pathway in Jurkat T cells, using a combination of phorbolester (PMA) and ionomycin, which mimic strong T-cell activation by activation of PKC family kinases and release of calcium from the ER, respectively. Unlike the wildtype form of MALT1, the E549A mutant was unable to promote PMA/ionomycin-induced activation of an NF-κB reporter gene (**Fig. 2B**). The activation of T-cells leads to the NF-κB-dependent production of the growth-promoting cytokine interleukin-2 (IL-2), which can be monitored at the transcriptional level using a dual IL-2 luciferase reporter assay. Transduction of Jurkat T-cells with wildtype MALT1, but not with the E549A mutant, led to a clear increase in IL-2 gene transcription induced by stimulation with PMA and ionomycin (**Fig. 2C**). Similar results were obtained when the cells were stimulated via the antigen receptor, using antigen presenting Raji B cells loaded with the bacterial superantigen staphylococcal enterotoxin E (SEE) (**Fig. 2D**). Thus, mutation of E549 within the dimerization interface of MALT1 renders the MALT1 enzyme biologically inactive.

**Figure 3:**
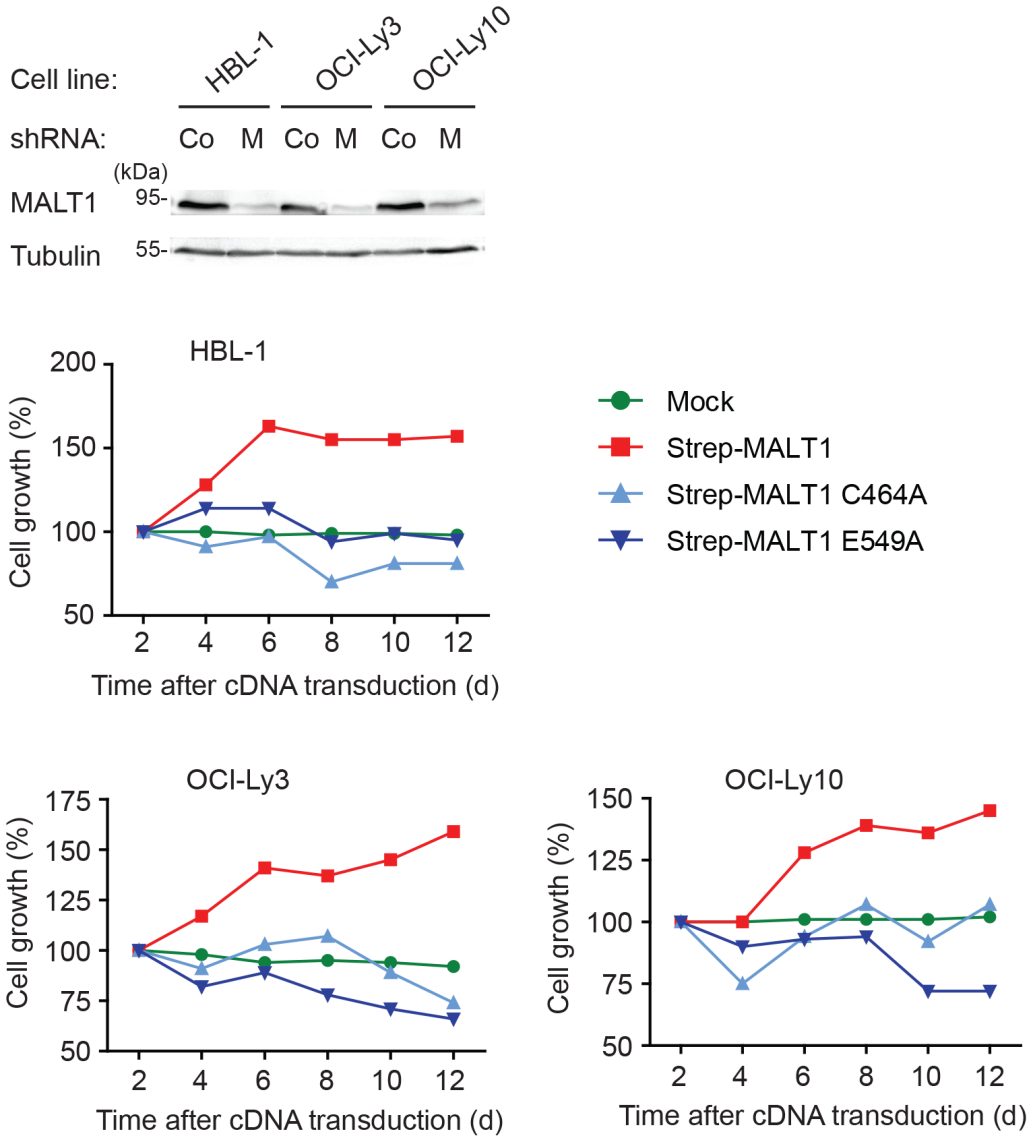
MALT1 E549 is required for survival of ABC DLBCL cell lines. The indicated ABC DLBCL cell lines were transduced with an inducible MALT1-specific shRNA and subsequently infected with constructs co-expressing GFP with Strep-MALT1, Strep-MALT1(E549A) or Strep-MALT1(C464A). Upon doxycycline-induced, shRNA-mediated silencing of endogenous MALT1, the cell viability of double infected GFP^+^ cells was monitored by flow cytometry over several days (lower panels). Efficient shRNA-mediated MALT1 silencing (M) was controlled by immunoblot (upper panels). As a negative control, we used a previously described *SC4* shRNA (Co) [52]. Results are representative of three independent experiments.

MALT1 has been reported to be constitutively active in ABC DLBCL as a consequence of oncogenic mutations in the B-cell receptor-associated CD79 chain or the CARMA1 protein [24], [25], [41], which act upstream of MALT1 in the signaling pathway leading from B-cell receptor triggering to NF-κB activation. The growth and survival of cells derived from ABC DLBCL is impaired upon silencing of MALT1 expression [23] or upon treatment of the cells with a MALT1 inhibitor [26], [27]. Moreover, impaired cellular survival has been reported for ABC DLBCL cell lines in which endogenous MALT1 is replaced by catalytically inactive MALT1 mutants [26], [34]. Therefore, we assessed the effect of the catalytically inactive mutant E549A on the survival of these cell lines under conditions of silencing of endogenous MALT1. In contrast to wildtype MALT1, both the C464A and the E549A mutant were unable to restore the growth of the ABC DLBCL cell lines, suggesting that E549-dependent formation of catalytically active MALT1 is essential for its oncogenic function (**Fig. 3**).

**Figure 4:**
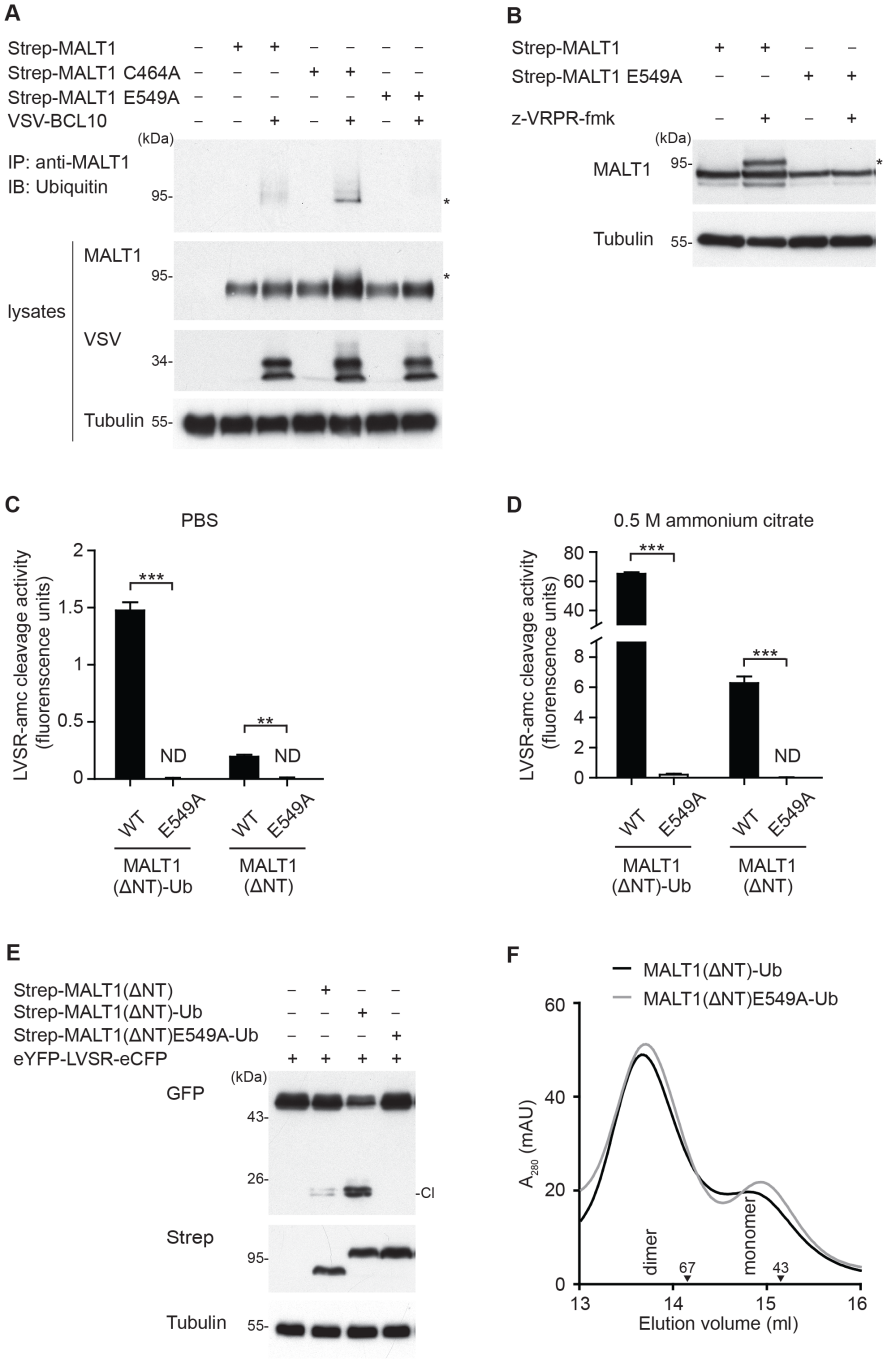
MALT1 E549 is required for MALT1 monoubiquitination. (A) Strep-tagged MALT1 wildtype or its E549A mutant were co-expressed with VSV-tagged BCL10 in HEK293T cells, and monoubiquitination of immunoprecipitated MALT1 was assessed by immunoblot. The asterisk (*) indicates monoubiquitinated MALT1. (B) Strep-tagged MALT1 wildtype or its E549A mutant were expressed in HEK293T cells, treated overnight with (+) or without (−) 100 µM of the MALT1 inhibitor z-VRPR-fmk, and MALT1 monoubiquitination was assessed by immunoblot. (C, D) The protease activity of the indicated recombinant purified proteins was determined using the fluorogenic MALT1 substrate LVSR-amc *in vitro* in PBS (C) or in the presence of 0.5 M ammonium citrate (D). Statistical significance is indicated by stars. *≤0.05, **≤0.01, ***≤0.005 (two-tailed Student’s *t*-test). (E) Indicated Strep-tagged MALT1 wildtype or E549A constructs were co-expressed with the reporter construct eYFP-LVSR-eCFP in HEK293T cells, and reporter cleavage was assessed by anti-GFP immunoblot (which detects both eCFP and eYFP). (F) Recombinant wildtype or E549A-mutant MALT1-Ub fusion proteins corresponding to the protease domain and C-terminal extension (aa 333–824, MALT1(ΔNT)-Ub) were analyzed by size exclusion chromatography in presence of PBS and 10% glycerol, and presented as the absorbance at 280 nm (A_280_) in milliarbitrary units’ (mAU). Downward arrowheads indicate the positions of the molecular weight standards ovalbumin (43 kDa) and bovine serum albumin (67 kDa). Data are representative of two (A, C, D and F) or three (B and E) independent experiments.

We have previously reported that MALT1 is modified by monoubiquitination in activated T cells, and that a construct mimicking C-terminal monoubiquitination of MALT1 (MALT1-Ub) is hyperactive, most likely as a consequence of its constitutive dimerization [34]. In contrast to recombinant unmodified MALT1, which becomes active upon addition of a kosmotropic salt *in vitro*, monoubiquitinated MALT1 is highly active, even in the absence of kosmotropic salt [34]. However, it has remained unknown how exactly monoubiquitination affects MALT1 activity. One possibility is that monoubiquitination is a prerequisite and thus an initiating trigger for subsequent dimerization. Alternatively, monoubiquitination may be the consequence of a dimerization that is initially induced via CBM complex formation or substrate binding. To gain insight into this question, we first assessed whether the E549A mutant was still able to undergo monoubiquitination. MALT1 monoubiquitination can be induced by co-expression of MALT1 with BCL10; it is further increased by the use of a catalytically inactive mutant of MALT1 (C464A) or by the pretreatment with the MALT1 inhibitor z-VRPR-fmk [34]. When tested under such experimental conditions, the E549A mutation was unable to undergo monoubiquitination (**Fig. 4** **, A and B**).

**Figure 5:**
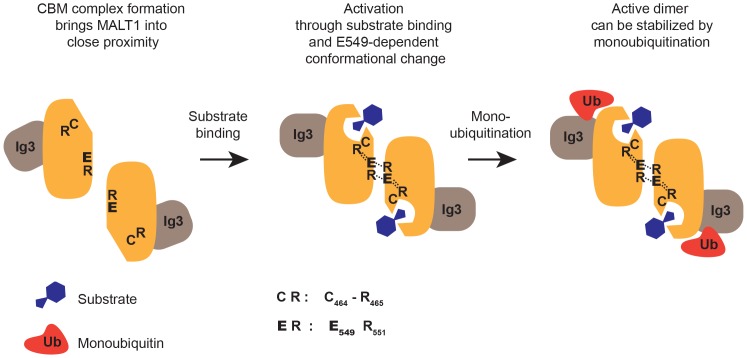
Hypothetical model of MALT1 activation. Upon antigen receptor activation, MALT1 may dimerize initially by an induced proximity mechanism that could be driven by assembly of the oligomeric CARMA1-BCL10-MALT1 (CBM) complex [43]. Upon substrate binding, E549 (highlighted in bold) in the dimerization interface most likely transmits a conformational change that strengthens dimerization. In this conformation, the active site C464 is reoriented via interaction of R465 with E549, and residues I550 and S552 of opposite subunits interact at the dimerization interface, as suggested by previous crystallographic studies [32], [33]. Our data support the idea that only the dimer in which the two subunits are correctly assembled in this E549-dependent manner can then be stabilized by monoubiquitination. Additional interactions between the α5 and α5′ helices of the two protease subunits that are thought to further stabilize the dimer interaction [33] are not shown in the model.

Next, we tested the catalytic activity and dimerization capacity of wildtype and E549A MALT1(ΔNT) constructs with or without C-terminally fused ubiquitin. First, we measured the activity of the corresponding recombinant purified proteins *in vitro*. The wildtype form of MALT1(ΔNT) was active already in PBS (**Fig. 4C**), and its activity could be further increased in the presence of 0.5 M ammonium citrate (**Fig. 4D**), as previously described [34]. In contrast, the corresponding E549A mutant showed no detectable activity, even in the presence of ammonium citrate (**Fig. 4** **, C and D**). C-terminal fusion of a ubiquitin moiety rendered the resulting wildtype MALT1(ΔNT)-Ub construct hyperactive, as previously described [34]. In contrast, C-terminal ubiquitination had only a minimal impact on the activity of the corresponding E549A-mutated MALT1(ΔNT)-Ub construct, which remained extremely low (**Fig. 4** **, C and D**). We also tested the activity of these constructs using a previously-described MALT1 activity reporter construct [34]. The construct is composed of enhanced yellow fluorescent protein (eYFP), followed by enhanced cyan fluorescent protein (eCFP), with an intervening short linker sequence containing a cleavage site (Leu-Val-Ser-Arg) previously identified in the MALT1 substrate RelB [10]. Cleavage of this reporter construct can be detected by immunoblot using an anti-GFP antibody that cross-reacts with both eCFP and eYFP. The wildtype form of the MALT1(ΔNT)-Ub construct efficiently cleaved this reporter, but mutation of E549 within the MALT1(ΔNT)-Ub construct led to a complete loss of proteolytic activity (**Fig. 4E**). However, when analyzed by size exclusion chromatography, both the wildtype and E549A-mutated forms of the MALT1(ΔNT)-Ub construct eluted preferentially as dimers (**Fig. 4F**). Collectively, these data suggest that an E549-dependent conformational change is a prerequisite for MALT1 monoubiquitination, which most likely serves to stabilize the active dimer conformation (**Fig. 5**).

## Discussion

In the present study, we have provided several lines of evidence that support a model in which E549 within the dimerization interface of MALT1 is required to promote the formation of a monoubiquitinated, catalytically active MALT1 conformation (**Fig. 5**). First, we showed that mutation of E549 affected dimerization of the isolated MALT1 protease domain *in vitro*, and inhibited the protease activity of MALT1. Second, we demonstrated that this mutant is impaired in its capacity to promote NF-κB activation and the transcription of the NF-κB target gene IL-2 in activated T cells. Third, we provided evidence for a role of E549 in promoting the MALT1-dependent growth of cells derived from ABC DLBCL, which are addicted to this signaling pathway. Finally, we showed that the E549A mutant was unable to undergo monoubiquitination, and that artificial C-terminal monoubiquitination was unable to overcome its activity defect.

How exactly does E549 affect the catalytic activity of MALT1? Our initial working hypothesis, based on a modeling approach predicting similar dimeric structures of the protease domains of caspases and MALT1, suggested that E549 and R551 are both located within the conserved β6 strand of the protease domain, which is the major determinant for the dimerization of caspases [42]. Our model further suggested that these residues might have a crucial role in stabilizing the protease-protease interface via electrostatic interactions. However, unlike the E549A mutant, the R551A mutant retained considerable enzymatic activity, arguing against a sole role for these two residues in stabilizing the protease dimer via an E549-R551 surface interaction. Indeed, additional interactions between I550 and S552 of opposing β6 strands, and interactions between the α5 and α5′ helices of the two protease subunits of MALT1 are thought to contribute to the stabilization of its dimeric conformation [33]. Our biochemical data support the idea that E549 contributes to MALT1 activation not simply by strengthening the dimer surface interactions, but also by promoting a dimer conformation that can be monoubiquitinated, which dramatically increases MALT1 activity [34]. This idea is compatible with previously published crystallographic structures of MALT1 that were obtained in the absence and presence of a peptide inhibitor acting as a substrate analog [32] (**Fig. S5, A and B**). In the absence of the MALT1 inhibitor (PDB code: 3V55 [32]), E549 forms a hydrogen bond with R551 of the same subunit, but not with R551′ of the opposing subunit (**Fig. S5A**). The crystal structures of MALT1 with the bound inhibitor (PDB code: 3V4O [32] and 3UOA [33]) suggest a major change in the hydrogen bonds formed by E549, and a crucial role for E549 in the stabilization of the active conformation of MALT1. In the substrate bound form, E549 forms a salt bridge with R465 that affects the position of the active site residue C464 [32], which is most likely stabilizing the active site conformation (**Fig. S5B**). The other changes provoked by substrate binding involve changes in the conformation of the R551 side chain that allow the formation of hydrogen bonds between R551 and E549 of opposing MALT1 monomers that further stabilize the dimeric structure [32] (**Fig. S5, B and C**). Thus, E549 may control the conformation of the active form of the dimeric protease domain both by its implication in a conformational change that renders the catalytic site active, and by strengthening the interactions between the subunits of the dimer (**Fig. S5C and** **Fig. 5**). Both of these mechanisms are impaired in our E549A mutant. The R551A mutant had a much weaker activity defect than the E549A mutant, which can be explained by R551 being involved in the stabilization of the conformation of the dimer but not in the formation of the active site. This hypothesis is consistent with findings of others, which have reported that an R551V mutant was still able to cleave the MALT1 substrate BCL10 [32]. In contrast, a R551E mutant introducing an additional negative charge in the dimerization interface that might affect the position of E549, had a profound impact on the activity of MALT1 [32]. The position changes of the active site residues and the R465, R551 and E549 residues are part of a cascade of conformational alterations that lead to a positional rearrangement of the the C-terminal Ig-like domain [32]. The E549A mutation hinders one of the first steps in this cascade and might consequently hamper the monoubiquitination process, for example by affecting the accessibility of the ubiquitination site K644 [34] or the interaction of the responsible (unknown) ubiquitin ligase with MALT1.

How does monoubiquitination affect the activation of MALT1? We have previously shown that addition of a C-terminal monoubiquitin moiety to MALT1 favors dimerization and activation of wildtype MALT1 [34]. In the case of the E549A mutant, such an artificial monoubiquitination did induce dimerization, but was not sufficient for MALT1 activation. Therefore, we propose a three-step model of MALT1 activation (**Fig. 5**), which includes as a first step an initial low affinity dimerization of MALT1 through induced proximity, as part of the CBM complex formation [43], and as a second step an E549-dependent conformational switch upon substrate binding, to allow formation of active dimers. Most likely, these active dimers need to be stabilized in a third step by monoubiquitination. In agreement with such a model, we observed that the E549A mutant was no longer monoubiquitinated, and that artificial C-terminal monoubiquitination of this mutant forced its dimerization, but was unable to overcome its activity defect. Thus, an E549-dependent conformational switch seems to be required for the subsequent stabilization of catalytically active MALT1 dimers by monoubiquitination (**Fig. 5**).

The data presented here and in our previous study [34] identify a unique mechanism of MALT1 activation that is clearly distinct from the activation of caspases, in which autoprocessing of the L2 and L2′ loops of the protease domains serves as a key event to stabilize the active conformation [4], [19], [21], [22]. Moreover, our findings support the idea that small molecule compounds specifically targeting the MALT1 dimerization interface could be useful as immunosuppressant or anti-lymphoma agents.

## Materials and Methods

### Modeling

Suitable templates for constructing a homology model of the caspase-like domain of MALT1 were identified using the HHpred method [44]. The model of the monomeric MALT1 protease domain was calculated based on the crystal structures of caspase-9, -3 and -8 (PDB codes 1NW9, 1CP3 and 2C2Z) sharing 19%, 18% and 18% sequence identity, respectively, with the MALT1 caspase-like domain. The dimeric MALT1 structure was calculated using the structure of caspase-8 (1F9E) as a template. Analogically to the mature/active structures of caspases the structure of MALT1 was modeled as cleaved after the R467 residue. The alignment (**Fig. S6**) and the modeling were done using the Modeller 9v5 program [45] and subsequent model energy minimization (200 steps of steepest descent minimization *in vacuo*) with the CHARMM package (version c34b) using the CHARMM22 all atom force field [46]. The Atomic Non-Local Environment Assessment (ANOLEA) potential [47] was employed to assess the quality of each of the structures and the conformation with the best overall score was chosen. The models were visualized using the UCSF Chimera software [48].

### Antibodies

Antibody against BCL10 (H197) was purchased from Santa Cruz Biotechnology, anti-Tubulin (B-5-1-2), anti-RelB (rabbit polyclonal) and anti-CYLD (D1A10) from Cell Signaling. Anti-FLAG (M2) and rabbit anti-VSV were from Sigma. Other antibodies used were anti-Strep-HRP (IBA BioTAGnology), anti-GFP (ALX 210-199; Enzo LifeSciences) and anti-Ubiquitin (P4D1; Covance). Affinity-purified MALT1 antibodies and antibodies specific for cleaved BCL10 have been previously reported [21], [49]. Horseradish peroxidase–coupled goat anti-mouse or anti-rabbit were from Jackson Immunoresearch.

### Plasmids

MALT1 point mutants were generated on pCR3-derived MALT1 expression constructs [21] by quick-change PCR using PfuUltra high-fidelity DNA polymerase AD (Agilent Technologies) and all mutations were verified by sequencing. The EYFP-LVSR-ECFP reporter construct, as well as the eukaryotic (pCR3-based) and bacterial (pGEX-based) MALT1-Ubiquitin constructs have been previously reported [34]. For silencing of MALT1 in DLBCL cell lines, cells were transduced with MALT1-specific shRNA (5′-GTCACAGAATTGAGTGATTTC-3′) as published [23]. MALT1 expression constructs resistant to shRNA-mediated silencing were subcloned into pMSCV-IRES-GFP [50]. The eYFP–Leu-Val-Ser-Arg–eCFP construct used to measure MALT1 activity and the lentiviral NF-κB reporter construct were generated as described [34].

### Transfection of Cells

Transient transfection of HEK293T cells and lentiviral transduction of Jurkat T cells have been previously described [21]. To transiently transfect Jurkat T cells by electroporation, 10^7^ cells were resuspended in 350 µl DPBS (Gibco) supplemented with CaCl_2_ and MgCl_2_ (100 mg/L each), and electroporated at 240 V, 950 µF using the GenePulser Xcell (Biorad) and 4 mm cuvettes (BTX).

### Cell Culture, Cell Stimulation and NF-κB Reporter Assays

HEK293T, Jurkat T cells and JTag cells were cultured in DMEM or in RPMI 1640 supplemented with 10% FCS and antibiotics, respectively. Lentivirally transduced Jurkat T cells were kept under puromycin selection (5 µg/ml) at all times. The ABC DLBCL cell lines HBL-1, OCI-Ly3 and OCI-Ly10 were cultured as described [26]. T cells were stimulated with either a mix of PMA (10 ng/ml; Alexis) and ionomycin (1 µM; Calbiochem) or with Raji cells presenting the superantigen SEE (streptococcal enterotoxin E; final concentration: 50 ng/ml). HEK293T cells were incubated with z-VRPR-fmk (100 µM) for 22 h. NF-κB or IL-2 dual luciferase reporter assays were performed as previously described [10].

### Lysis, Protein Precipitation and Immunoblotting

Cells were lysed in lysis buffer containing 50 mM HEPES pH 7.5, 150 mM NaCl, 1% Triton-X-100, protease inhibitors (Complete; Roche) and phosphatase inhibitors (NaF, Na_4_P_2_O_7_ and Na_3_VO_4_). After preclearing the lysates with sepharose beads for 20 min, StrepTactin sepharose beads (IBA) were added and samples were incubated for 30 min at 4°C. The samples were then washed three times with lysis buffer and once with Tris-NaCl buffer (20 mM Tris pH 7.4, 150 mM NaCl). Where indicated, some samples were lysed with 1% SDS. After quenching the samples in a 1∶10 ratio with lysis buffer, Strep-MALT1 was recovered by precipitation with anti-MALT1 antibody coupled to protein G sepharose. Samples were boiled in reducing SDS sample buffer and subjected to SDS-PAGE and immunoblot as described [21].

### Protein Purification and Analysis by FPLC

Recombinant GST proteins containing the MALT1 caspase-like domain only, the caspase-like domain and the C-terminus (MALT1(ΔNT)), or the same construct fused to ubiquitin Ub (MALT1(ΔNT)-Ub), mutated or not at E549, were generated and purified as previously described [21]. For gel filtration analysis, IPTG-induced BL21 bacteria were lysed in a buffer containing 50 mM HEPES, pH 7.9, 300 mM NaCl, 1 mM EDTA, 0.1% NP-40, 2 mM DTT, protease inhibitors (Complete; Roche) and 5% glycerol. After lysis, glycerol concentration was increased to 10% and GST proteins were immobilized for 2.5 h at 4°C on glutathione-Sepharose beads (GE Healthcare). After extensive washing, beads were incubated for 2.5 h at 4°C with PreScission enzyme (GE Healthcare) in wash buffer containing 5% glycerol. Purified proteins were subjected to gel filtration performed on an ÄKTA FPLC system at 4°C using a Superdex 200 column (GE Healthcare) in PBS with 10% glycerol (pH 7.4) with a flow rate of 0.35 ml/min.

### *In vitro* Protease Activity Assay

To measure protease activity *in vitro*, recombinant or precipitated proteins were incubated with 50 µM of the fluorescent substrate Ac-LVSR-amc (Peptides International) for 4 h at 30°C, while detecting the protease activity of MALT1 with a Synergy microplate reader (BioTek).

### Retroviral Transduction and MALT1 shRNA Rescue

For efficient retroviral transductions, cell lines were engineered to express the murine ecotropic receptor as previously described [23]. Additionally, these cell lines were engineered to express the bacterial tetracycline repressor allowing doxycycline-inducible shRNA expression [23]. For shRNA induction, doxycycline (20 ng/ml) was used [23], [51]. For the MALT1 rescue experiment, cells were stably transduced with the MALT1 shRNA. The MALT1 shRNA-positive cells were subsequently transduced to constitutively express GFP together with Strep-MALT1 wildtype, Strep-MALT1(E549A), or Strep-MALT1(C464A). In order to induce knockdown of the endogenous MALT1 and not the exogenously expressed Strep-MALT1 variants, the shRNA binding sites in these constructs were mutated at cDNA position 313 from GAATTG to GAGTTA, without changing the encoded amino acids. The double-transduced cells were monitored for live GFP^+^ cells using flow cytometry as previously described [10].

### Statistical Analysis

Two-tailed Student’s *t*-test was used for statistical analysis; *P* values ≤0.05 were considered statistically significant.

## Supporting Information

Figure S1 **Comparison of the modeled and crystallographic structure of the MALT1 caspase-like domain.** Superposition of the homology model and the inhibitor bound crystal structure of MALT1 (3UOA.pdb) [33] with the color-coded RMSD values calculated over Cα atoms. The colors used indicate different RMSD values; red: over 4 Å, blue: 2–4 Å, green: below 2 Å. The crystal structure is shown in transparent white.
(TIF)

Figure S2 **The dimerization interface in the homology model of MALT1.** Model of the MALT1 protease domain, calculated based on the crystal structures of caspase-9, -3 and -8 (PDB codes 1NW9, 1CP3 and 2C2Z, respectively) and the dimeric structure of caspase-8 (1F9E). One of the dimer subunits is shown in beige, the other one in green. Side chains of residues C464, R465, E549 and R551 are shown in ball and stick representation, with nitrogen atoms in blue and oxygen atoms in red. Predicted hydrogen bonds between E549 and R551, and between R465 and E549, are indicated (dashed lines). The figure was prepared using Chimera software [48].
(TIF)

Figure S3 **Mutation of Arg551 into Ala partially impairs MALT1 catalytic activity** ***in vitro.*** The activity of the indicated Strep-tagged MALT1 wildtype (WT) and R551A mutant construct, precipitated from transfected HEK293T cells, was determined *in vitro* in presence of 1 M ammonium citrate using the MALT1 substrate LVSR-amc. Protein levels in lysates and precipitations were controlled by immunoblot. Left margin, molecular size marker in kilodalton (kDa). Black lines indicate where lanes have been removed. PD: pull-down.
(TIF)

Figure S4 **Mutation of Glu549 into Ala does not affect binding of MALT1 to BCL10.** To assess whether mutation of Glu549 into alanine (E549A) affects the binding of MALT1 to BCL10, HEK293T cells were transfected with the indicated combinations of Flag-tagged BCL10 and Strep-tagged MALT1 wildtype, or E549A expression constructs, and StrepTactin precipitates and cell lysates were blotted with the indicated antibodies. PD: pull-down.
(TIF)

Figure S5 **The dimerization interface in the crystal structures of the free and inhibitor-bound forms of MALT1.** (A) Crystal structure of the dimeric MALT1 protease domain in the absence of the MALT1 inhibitor (PDB code: 3V55) [32]. Zoom shows dimerization interface and the position of the catalytic site cysteine residue of one subunit. Side chains of residues C464, R465, E549 and R551 are shown in ball and stick representation, with nitrogen atoms in blue and oxygen atoms in red. Hydrogen bonds between side chains of E549 and R551 residues are represented with dashed lines. The figures were prepared with Chimera software [48]. (B) Crystal structure of the dimeric MALT1 protease domain in the presence of the MALT1 inhibitor z-VRPR-fmk (PDB code: 3V4O) [32]. The inhibitor is shown in dark green. Zoom shows dimerization interface and the position of the catalytic site cysteine residue of one subunit. Residues C464, R465, E549 and R551 are shown in ball and stick representation, with nitrogen atoms in blue and oxygen atoms in red. The hydrogen bonds between the side chains of E549 and R551 and between R465 and E549 are indicated with dashed lines. The figures were prepared with Chimera software [48]. (C) Superposition of the dimerization interfaces shown in (A) and (B). Black arrows indicate movements of the side chains in response to inhibitor binding.
(TIF)

Figure S6 **Alignment of the protease sequences used for modeling.** Alignment of the MALT1 caspase-like domain and caspase-3, -8, -9 sequences used for building the MALT1 homology model. The coloring scheme used for conserved residues in the alignment is indicated; green: polar amino acids, blue: hydrophobic amino acids, red: basic amino acids, magenta: acidic amino acids, cyan: aromatic amino acids, orange: Gly residues, yellow: Pro residues, pink: Cys residues. Alignment was visualized with Jalview [53].
(TIF)
