# Supplementary figures and images for: Monoubiquitination and Activity of the Paracaspase MALT1 Requires Glutamate 549 in the Dimerization Interface
Source: PLoS One. 2013 Aug 19;8(8):e72051. doi: 10.1371/journal.pone.0072051 (PMC3747146; doi:10.1371/journal.pone.0072051)

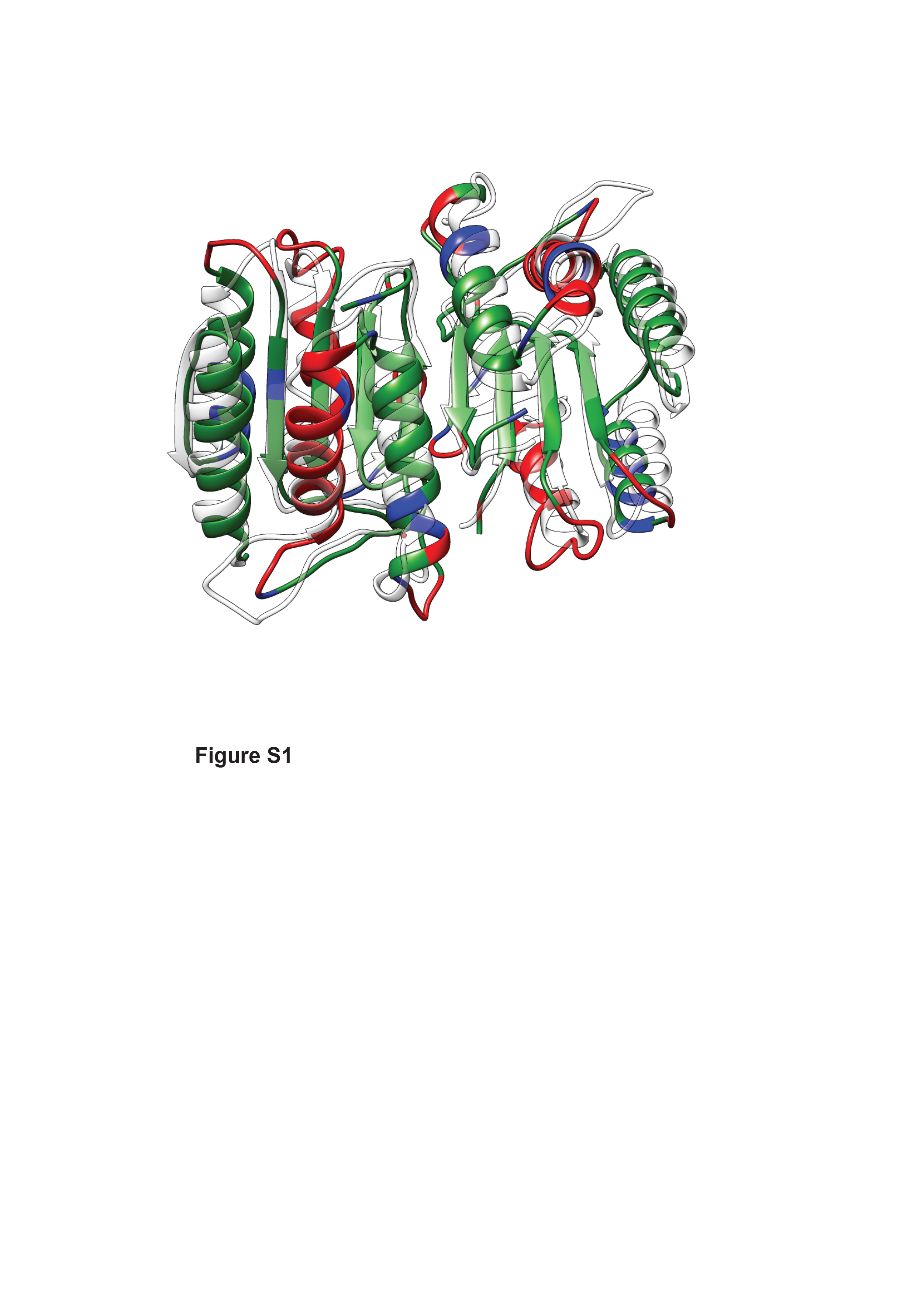

Supplement: Figure S1 — Comparison of the modeled and crystallographic structure of the MALT1 caspase-like domain. Superposition of the homology model and the inhibitor bound crystal structure of MALT1 (3UOA.pdb) [33] with the color-coded RMSD values calculated over Cα atoms. The colors used indicate different RMSD values; red: over 4 Å, blue: 2–4 Å, green: below 2 Å. The crystal structure is shown in transparent white. (TIF) [file pone.0072051.s001.tif]

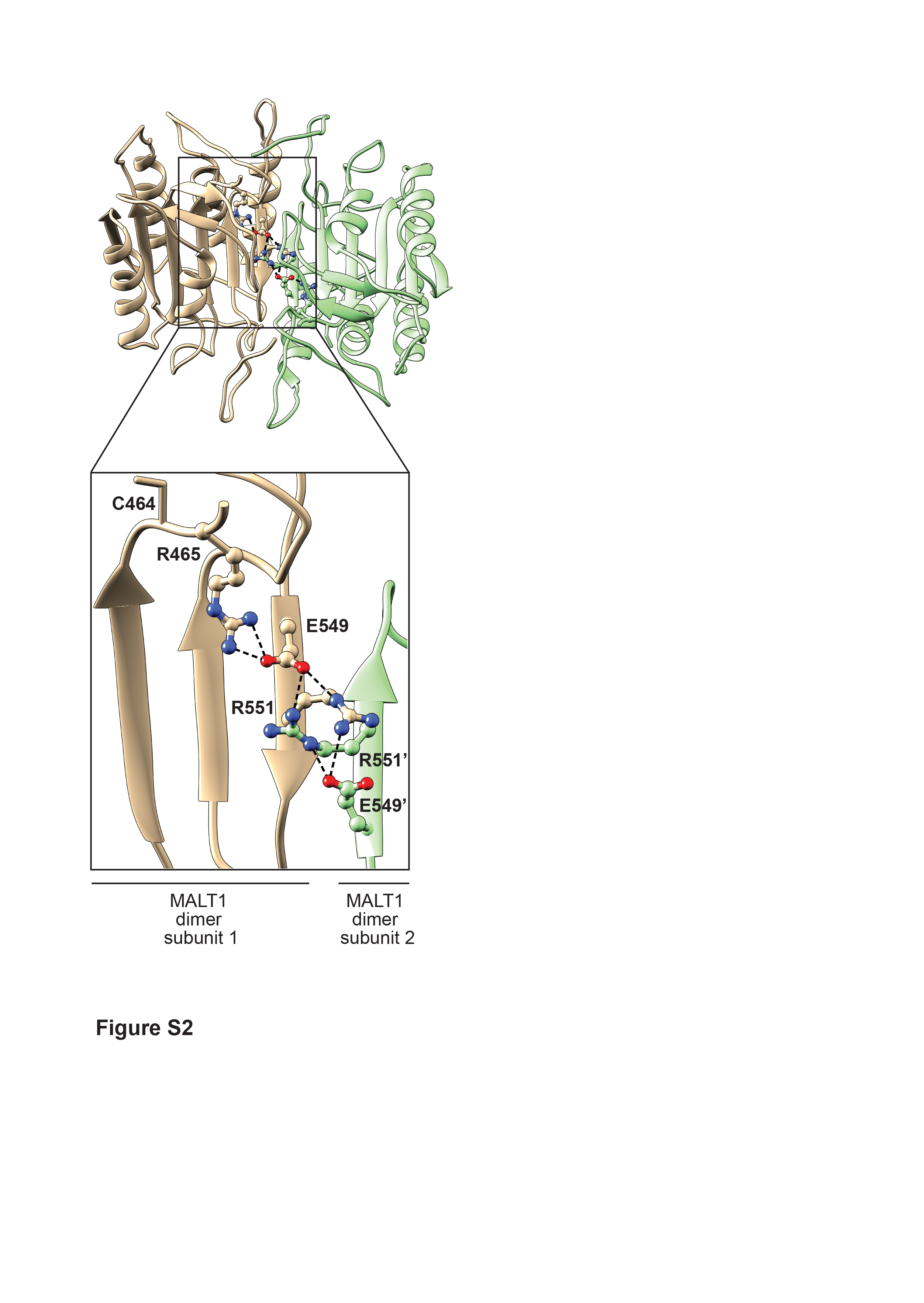

Supplement: Figure S2 — The dimerization interface in the homology model of MALT1. Model of the MALT1 protease domain, calculated based on the crystal structures of caspase-9, -3 and -8 (PDB codes 1NW9, 1CP3 and 2C2Z, respectively) and the dimeric structure of caspase-8 (1F9E). One of the dimer subunits is shown in beige, the other one in green. Side chains of residues C464, R465, E549 and R551 are shown in ball and stick representation, with nitrogen atoms in blue and oxygen atoms in red. Predicted hydrogen bonds between E549 and R551, and between R465 and E549, are indicated (dashed lines). The figure was prepared using Chimera software [48]. (TIF) [file pone.0072051.s002.tif]

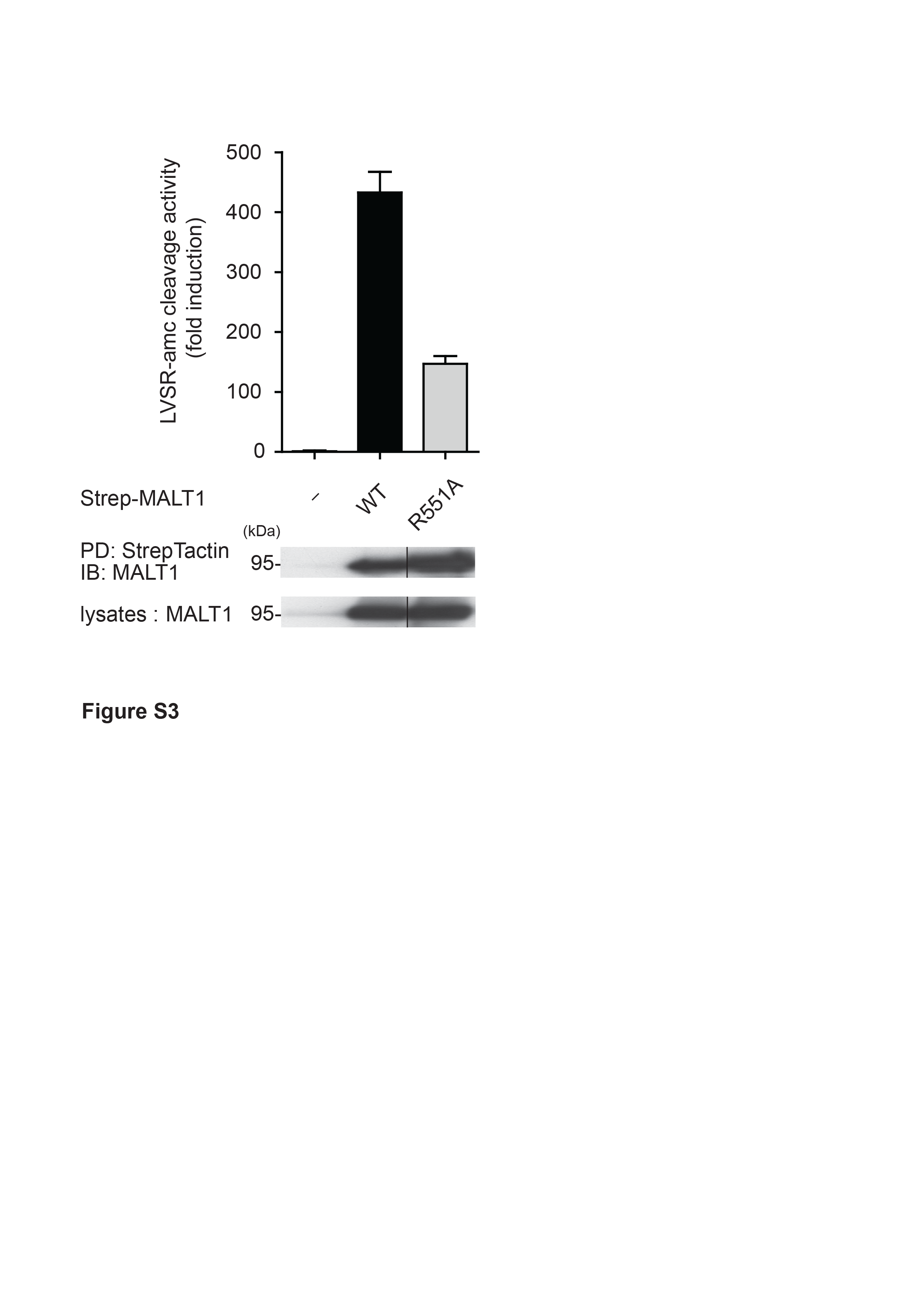

Supplement: Figure S3 — Mutation of Arg551 into Ala partially impairs MALT1 catalytic activity in vitro. The activity of the indicated Strep-tagged MALT1 wildtype (WT) and R551A mutant construct, precipitated from transfected HEK293T cells, was determined in vitro in presence of 1 M ammonium citrate using the MALT1 substrate LVSR-amc. Protein levels in lysates and precipitations were controlled by immunoblot. Left margin, molecular size marker in kilodalton (kDa). Black lines indicate where lanes have been removed. PD: pull-down. (TIF) [file pone.0072051.s003.tif]

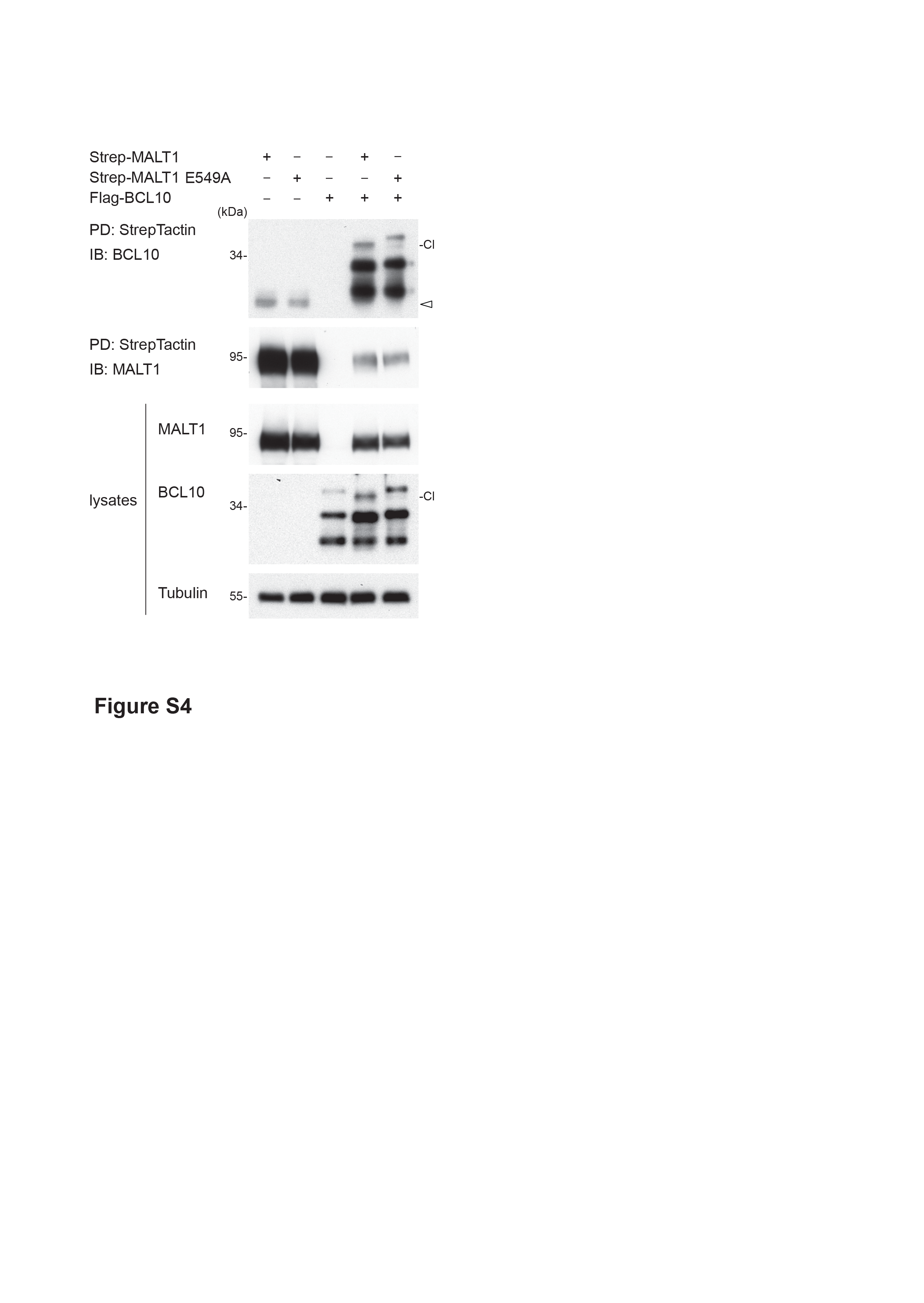

Supplement: Figure S4 — Mutation of Glu549 into Ala does not affect binding of MALT1 to BCL10. To assess whether mutation of Glu549 into alanine (E549A) affects the binding of MALT1 to BCL10, HEK293T cells were transfected with the indicated combinations of Flag-tagged BCL10 and Strep-tagged MALT1 wildtype, or E549A expression constructs, and StrepTactin precipitates and cell lysates were blotted with the indicated antibodies. PD: pull-down. (TIF) [file pone.0072051.s004.tif]

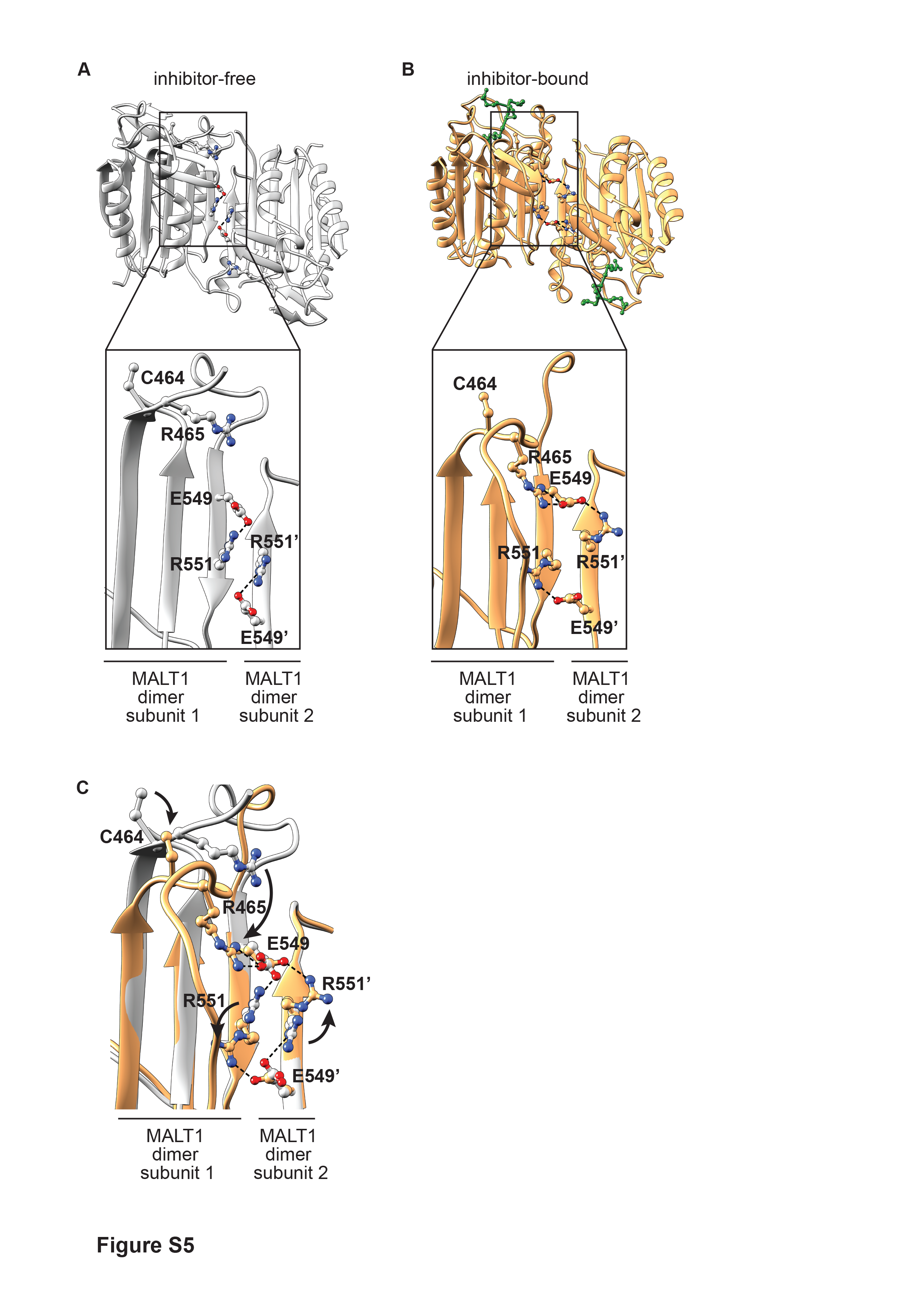

Supplement: Figure S5 — The dimerization interface in the crystal structures of the free and inhibitor-bound forms of MALT1. (A) Crystal structure of the dimeric MALT1 protease domain in the absence of the MALT1 inhibitor (PDB code: 3V55) [32]. Zoom shows dimerization interface and the position of the catalytic site cysteine residue of one subunit. Side chains of residues C464, R465, E549 and R551 are shown in ball and stick representation, with nitrogen atoms in blue and oxygen atoms in red. Hydrogen bonds between side chains of E549 and R551 residues are represented with dashed lines. The figures were prepared with Chimera software [48]. (B) Crystal structure of the dimeric MALT1 protease domain in the presence of the MALT1 inhibitor z-VRPR-fmk (PDB code: 3V4O) [32]. The inhibitor is shown in dark green. Zoom shows dimerization interface and the position of the catalytic site cysteine residue of one subunit. Residues C464, R465, E549 and R551 are shown in ball and stick representation, with nitrogen atoms in blue and oxygen atoms in red. The hydrogen bonds between the side chains of E549 and R551 and between R465 and E549 are indicated with dashed lines. The figures were prepared with Chimera software [48]. (C) Superposition of the dimerization interfaces shown in (A) and (B). Black arrows indicate movements of the side chains in response to inhibitor binding. (TIF) [file pone.0072051.s005.tif]

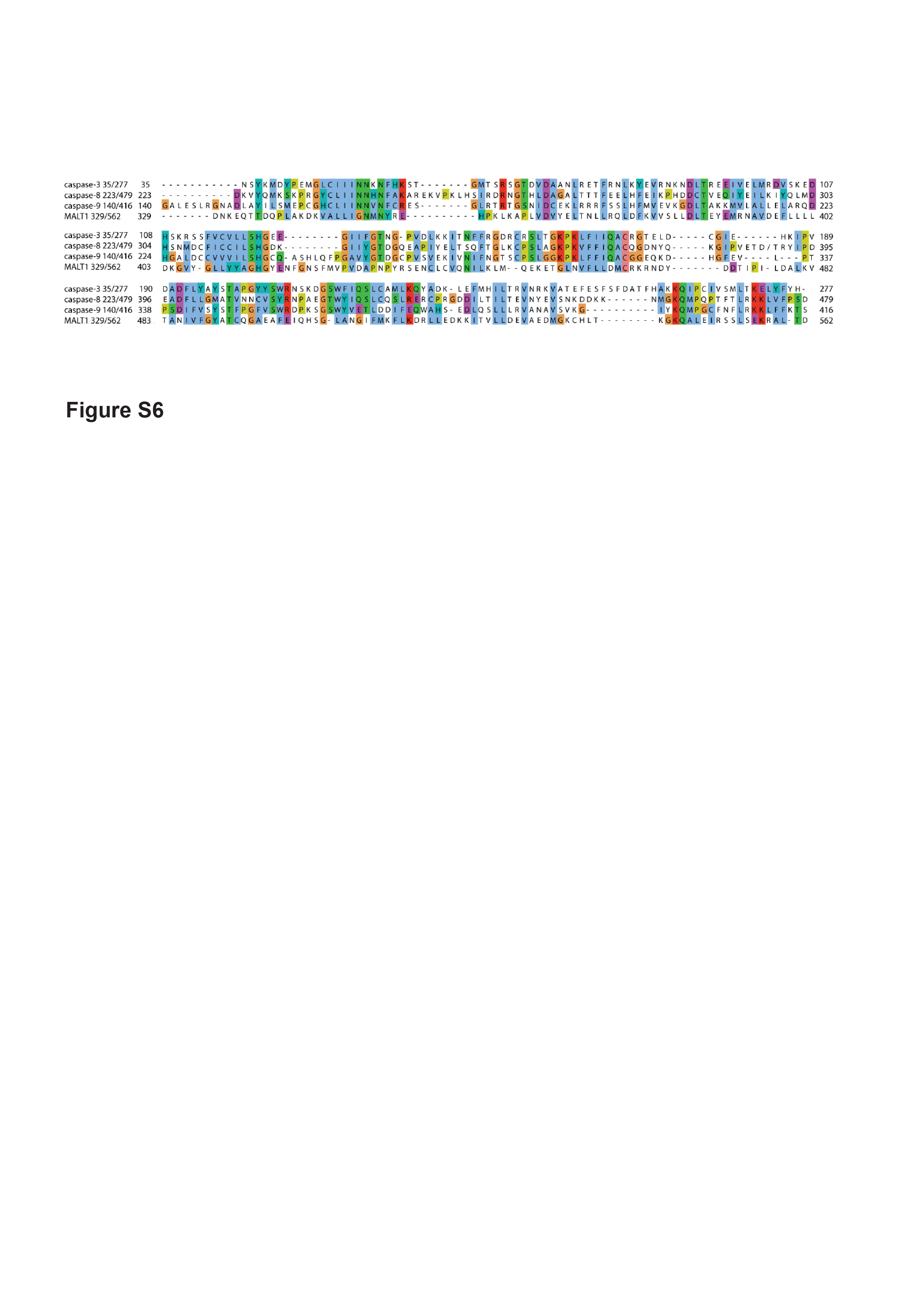

Supplement: Figure S6 — Alignment of the protease sequences used for modeling. Alignment of the MALT1 caspase-like domain and caspase-3, -8, -9 sequences used for building the MALT1 homology model. The coloring scheme used for conserved residues in the alignment is indicated; green: polar amino acids, blue: hydrophobic amino acids, red: basic amino acids, magenta: acidic amino acids, cyan: aromatic amino acids, orange: Gly residues, yellow: Pro residues, pink: Cys residues. Alignment was visualized with Jalview [53]. (TIF) [file pone.0072051.s006.tif]
